# Multi-omics integrative analysis with genome-scale metabolic model simulation reveals global cellular adaptation of *Aspergillus niger* under industrial enzyme production condition

**DOI:** 10.1038/s41598-018-32341-1

**Published:** 2018-09-26

**Authors:** Hongzhong Lu, Weiqiang Cao, Xiaoyun Liu, Yufei Sui, Liming Ouyang, Jianye Xia, Mingzhi Huang, Yingping Zhuang, Siliang Zhang, Henk Noorman, Ju Chu

**Affiliations:** 1 State Key Laboratory of Bioreactor Engineering, East China University of Science and Technology, Shanghai, 200237 P. R. China; 2 DSM Biotechnology Center, P.O. Box 1, 2600MA Delft, The Netherlands

## Abstract

Oxygen limitation is regarded as a useful strategy to improve enzyme production by mycelial fungus like *Aspergillus niger*. However, the intracellular metabolic response of *A. niger* to oxygen limitation is still obscure. To address this, the metabolism of *A. niger* was studied using multi-omics integrated analysis based on the latest GEMs (genome-scale metabolic model), including metabolomics, fluxomics and transcriptomics. Upon sharp reduction of the oxygen supply, *A. niger* metabolism shifted to higher redox level status, as well as lower energy supply, down-regulation of genes for fatty acid synthesis and a rapid decrease of the specific growth rate. The gene expression of the glyoxylate bypass was activated, which was consistent with flux analysis using the *A. niger* GEMs iHL1210. The increasing flux of the glyoxylate bypass was assumed to reduce the NADH formation from TCA cycle and benefit maintenance of the cellular redox balance under hypoxic conditions. In addition, the relative fluxes of the EMP pathway were increased, which possibly relieved the energy demand for cell metabolism. The above multi-omics integrative analysis provided new insights on metabolic regulatory mechanisms of *A. niger* associated with enzyme production under oxygen-limited condition, which will benefit systematic design and optimization of the *A. niger* microbial cell factory.

## Introduction

With a GRAS (generally regarded as safe) status, *Aspergillus niger* is widely applied in the biosynthesis of organic acids and enzymes^1^. *A. niger* has an excellent ability of protein expression and secretion, such as for the industrial production of glucoamylase^2^. In 2007, the genome sequence and annotation information of *A. niger* was published^3^, which became an important basis for systems biology studies of *A. niger*. Genome annotation of *A. niger* showed its huge potential as an efficient cell factory^3^ for the production of different enzymes and secondary metabolites. A genome-scale metabolic model (GEMs) was also reconstructed based on the genome annotation of *A. niger*^4^. Recently, the GEMs of *A. niger* was further updated in our lab^5^. Centering on enzyme and protein production, separate studies on *A. niger* metabolomics, transcriptomics and proteomics have been conducted^6^.

In the industrial enzyme production by *A. niger*, the poor solubility of oxygen limits mass transfer in bioreactors. This is further aggravated by the complex mycelial morphology, resulting in oxygen limitation for the cell metabolism. As the growth of *A. niger* is strictly aerobic^7^, a limited oxygen supply has a strong impact on the fermentation process. For citric acid production, the limited oxygen supply leads to a significant increase in productivity^8^. Although the specific production rate of glucoamylase was decreased due to the oxygen limitation, its yield per unit of substrate was increased^2^. The similar results could be found in exogenous protein production by *Pichia pastoris*^9^ and other microorganisms. It has been found that large amounts of organic acids (like oxalic acid and citric acid) and polyols (like mannitol and erythritol) were secreted by *A. niger* under oxygen limited conditions^10,11^, indicating a high intracellular redox level. These microbial physiology phenomena indicate that the metabolic balance between cell growth and product synthesis is sensitive to oxygen limitation in *A. niger*. However, details of the global metabolic changes and their interpretation in terms of regulatory mechanisms has only been subject of little systematic research.

Omics studies play an increasingly important role in investigation of the cell metabolic response and regulation mechanisms, and there are a few studies using these approaches to study how cells adapt to oxygen limited conditions. Via transcriptome analysis of *Trichoderma reesei*, it was found that the expression of genes from metabolic pathways related to the energy consumption were significantly down-regulated in response to the limited oxygen supply^12^. Using proteomics analysis, it was shown that the expression of 117 proteins in *A. fumigatus*, involved in the PP pathway, TCA pathway and EMP pathway, was up-regulated to adapt to the hypoxic environment^13^. With the aid of transcriptome analysis, Choi *et al*.^14^ found that the genes involved in the sterol regulatory synthesis pathways were activated under oxygen limited conditions, which facilitated the synthesis of sterols and maintained the cell mycelial growth capabilities.

Compared with single omics analysis, a multi-omics integrative analysis could help to reveal interactions among different metabolic regulation levels. Based on the evidence from transcriptome analysis and molecular experiments, Kroll *et al*.^15^ found that the electron transport chain plays an important role in sensing the extracellular oxygen concentration and transmitting the hypoxia signal to the mitochondria. The metabolic characteristics of glucoamylase production by integration of ^13^C metabolic flux analysis and metabolomics^16^ was recently carried out in our lab. The results showed that the intracellular metabolic fluxes were redistributed to respond the enzyme synthesis and redox balance. Baumann *et al*.^17^ studied the metabolic mechanism of *Pichia pastoris* under oxygen limited conditions with integrative analysis of metabolomics, transcriptomics and proteomics, and found that flux changes in the PP, TCA and EMP pathways were mainly regulated at a transcriptional level.

To better understand the mechanisms supporting a high yield of glucoamylase production and global metabolic regulation under oxygen limitation, the multi-omics integrative analysis based on GEMs is employed, which provides holistic views for the rational optimization of industrial bioprocess and strain performance.

## Materials and Methods

### Strains and cultivations

The glucoamylase high-producing strain *Aspergillus niger* DS03043, donated by DSM (Netherlands) was used in all the cultivations in this work. To obtain spores, Petri dishes containing PDA (Potato Dextrose Agar) medium were incubated with spores from a frozen stock (stored in 50% glycerin at −80 °C). During seed culture, 500 mL shake flasks with baffles were inoculated with 10^7^ spores per 100 mL broth. A 5 L fermentor with an electronic balance was used for the fed-batch cultivations with the agitation rate at 375 rpm and the aeration at 1 vvm. During the cultivation, the overpressure was maintained at 0.05 MPa and the temperature was at 34 °C. The broth pH was maintained at 4.5 by addition of NH_3_ solution (5% w/w). The working volume for the 5 L fermenter during batch cultivation was 3 L. When the glucose concentration reduced to 5 g/L (after about 36 h of the fermentation) during the batch cultivation, the feed was started and the glucose concentration was kept at around 5 g/L by adjusting the feed rate. Concentrations of oxygen and carbon dioxide in the exhaust gas were determined by process mass spectrometers (MAX300-LG, Extrel) during the fermentation and the dissolved oxygen concentration in the broth was determined with a low-drift polarographic electrode (Mettler Toledo).

The medium for the seed and fed-batch fermentations can be found in the literature reported by Lu^18^.

### Quantification of biomass and enzyme activity

10 mL fermentation broth was filtered by filter paper, pre-weighed and pre-dried to a constant weight (at 80 °C for 24 h). Biomass was rinsed three times with deionized water and dried at 80 °C for 24 h. Dried biomass was re-weighed immediately. Enzyme activity of all samples was determined by a standard procedure^18^.

### Quantification of extracellular sugar and organic acids

Residual sugar from the fermentation broth was determined by a glucose analyser (Shandong Academy of Sciences, China). Extracellular organic acids (acetic acid, citric acid, oxalic acid, malic acid, fumaric acid, pyruvic acid and succinic acid) were determined by high performance liquid chromatography (HPLC). The HPLC was equipped with an ion exclusion column and an absorption detector spectrophotometer. 10 mM H_2_SO_4_ was used to wash the ion exclusion chromatography column with the flow rate of 0.5 mL/min at 50 °C and the wavelength of the spectrophotometer was set at 210 nm.

### Sampling and quantitative analysis of intracellular metabolites

The protocol for quantitative analysis of intracellular metabolites was modified based on Douma *et al*.^19^. Using fast sampling equipment, 1–2 ml broth was pumped from the 5 L fermenter into a 10 ml precooled quenching solution (40% v/v methanol solution at −27.6 °C) at 18 h, 24 h, 36 h, 48 h, 60 h, 72 h and 96 h, respectively. The tubes were weighed before and after the sampling procedure to estimate the exact amount of broth. Then, extracellular metabolites were removed by vacuum filtration and filter cake was washed by 120 ml precooled quenching solution. Isotope dilution mass spectrometry (IDMS)^20^ was used in this work for the quantification of metabolite concentrations. Washed filter cake, as well as ^13^C internal standard solution was added to 25 ml pre-warmed 75% (v/v) ethanol solution and the extraction continued for 3 minutes at 95 °C. The metabolites concentration was determined with UPLC-MS/MS (Thermo Fisher Scientific Corporation) and GC-MS.

As for metabolomics data, the principal component analysis (PCA) and partial least square discriminant analysis (PLS-DA) were conducted based on the R programming language. If the variable importance of the projection (VIP) score of one metabolite is above 1, it means that the pool size of this metabolite changes significantly in different fermentation phases. The Heatmap analysis of the metabolomics data from different fermentation phases was conducted using the superheat package of the R language (https://cran.r-project.org/web/packages/superheat/).

### Transcriptome analysis

According to the online DO profile, the sampling time for RNA-seq analysis was set at 18 h, 24 h, 42 h and 66 h, which corresponding to the logarithmic phase and early, middle, late phases of oxygen limitation, respectively. After sampling, the broth was immediately frozen in liquid nitrogen and stored at −80 °C. These frozen samples were sent to Sangon Biotech for RNA extraction and RNA samples were sent to the Beijing Genomics Institute (BGI) for sequencing. Qualified RNA samples of each sampling time were ensured with at least 2 replicate samples for sequencing. Gene expression data of different phases was clustered by the Mfuzz package based on R language^21^. The DAVID database was used for GO enrichment analysis of the interested gene sets^22^. The Piano package based on the R language was used for KEGG pathway and GO function enrichment analysis of differentially expressed genes^23^. For gene set analysis, a mapping, established between genes of *A. niger* and the KEGG pathway and the GO function, as well as gene expression data of different phases, were used as inputs of the Piano package for statistical analysis. For more details, please refer to the literature^23^.

### Flux simulation using GEMs

Flux balance analysis (FBA) based on constraints is widely exploited in the fields of genome-scale metabolic network reconstruction and cellular phenotypic prediction^24^. FBA is used for prediction and analysis of intracellular fluxes with the optimization of an objective function under constraints. Constraints^25^ could be an intracellular metabolite balance (Equation 2), reaction reversibility, a maximum enzyme reaction rate and an exchange reaction rate (Equation 3). An objective function is for example maximization of cell growth or optimization of cell energy utilization^26^ (Equation 1). The rate of each reaction in the model has a limit. For reversible reactions, the upper and lower bound is set to 1000 and −1000 mmol/g_Biomass_.h, respectively. For irreversible reactions, the lower bound is set to zero. The COBRA toolbox and the Gurobi 5 linear optimization algorithm were used for FBA analysis in this study.

1 $${\rm{Objective}}\,{\rm{function}}:\,{\rm{\max }}/{\rm{\min }}\,{\rm{Z}}={{\rm{C}}}^{{\rm{T}}}\,\ast \,{\rm{v}}$$

2 $${\rm{Constraints}}:{\rm{S}}\,\ast \,{\rm{v}}={\rm{0}}$$

3 $${\rm{lb}}\le {\rm{v}}\le {\rm{ub}}$$

where S is a m × n sparse matrix, in which m refers to the number of metabolites and n refers to the number of reactions. v represents the rate vector of all reactions. lb and ub defines the lower and upper bound of each reaction, respectively. In equation 1, C^T^ refers to the coefficient of each metabolite in the objective function.

Intracellular metabolic fluxes were predicted by the parsimonious FBA (pFBA)^27^. The model used in this study, *A. niger* GEMs iHL1210 ^5^ that was updated recently by our laboratory, contains 1727 mass and proton balanced reactions and 1210 ORFs (see Supplementary File 3). Maximization of cell growth was set as the objective function and the measured values of q_S_, q_by-product_, q_P_, q_O2_ and m_ATP_ as constraints during simulation for the fed-batch fermentations (see Supplementary File 3). The prediction performance using iHL1210 was assessed by comparing the predicted values of μ and q_CO2_ with the measured values.

## Results

**Figure 1:**
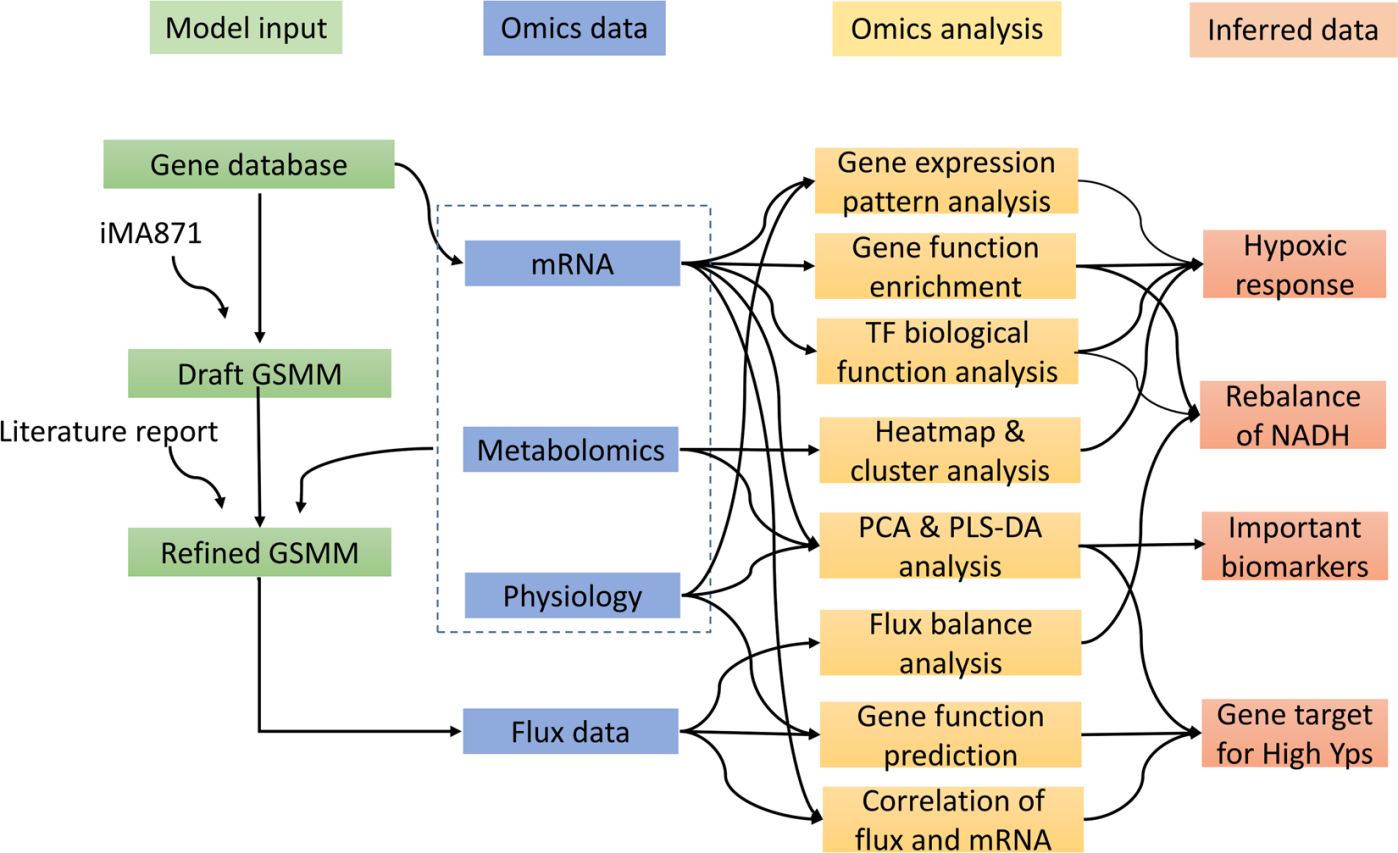
Framework of multi-omics integration analysis used in this work.

In order to investigate the global regulating mechanisms of cell metabolism under oxygen-limited condition, the integrative analysis of physiological phenotypic data, metabolomics, transcriptomics and fluxomics was adopted in this work (Fig. 1).

### Quantitative analysis of physiological parameters

**Figure 2:**
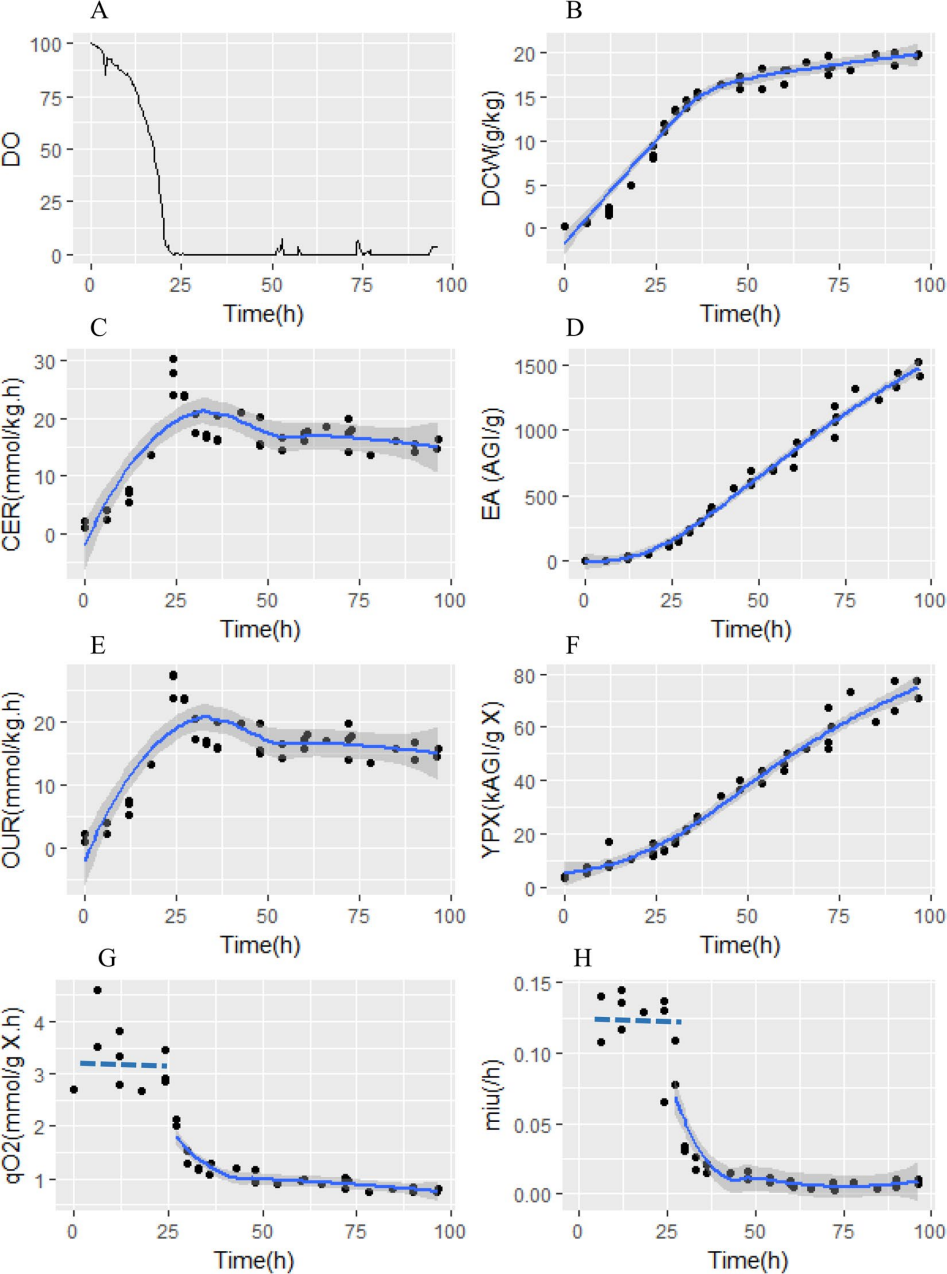
Profiles of DO (**A**), dry cell weight (DCW) (**B**), CO_2_ production rate (CER) (**C**), glucoamylase enzyme activity (**D**), O_2_ uptake rate (OUR) (**E**), yield of glucoamylase per gram biomass (Y_PX_) (**F**), specific oxygen uptake rate (q_O2_) (**G**) and specific growth rate (µ) (**H**) for *A. niger* DS03043 with high glucoamylase production during fed-batch cultivations.

Mimicking the industrial production using *A. niger*, fed-batch cultivations applying oxygen limited strategy were conducted in this work. The fed-batch fermentation process could be initially divided into 2 main phases, i.e. aerobic phase (0–20 h) and oxygen limited phase (20–72 h) according to profiles of the dissolved oxygen concentration (DO) (Fig. 2A). The changes in the profiles of oxygen uptake rate (OUR), carbon dioxide emission rate (CER) and dry cell weight (DCW) were determined by oxygen supply (OTR). When the oxygen supply was limited, the specific growth rate (μ) quickly decreased (Fig. 2H). Meanwhile, the OUR and CER decreased sharply to a stable level (Fig. 2C and E). By-products analysis showed that organic acids and polyols were slightly excreted by cell (see Supplementary File 1, Fig. S1). The detailed calculation showed that the total carbon ratio of these by-products (Y_by-prodcuts/s_) is about 5%, thus it can be concluded that most of carbon source fluxed into the biomass, CO_2_ and product. During the oxygen limited phase, in contrast to the increased yield of glucoamylase (Fig. 2F), μ was decreased continuously and lower than 0.02 h^−1^ (Fig. 2H) at the end of fermentation.

### Profiling of key metabolites in core carbon metabolism

**Figure 3:**
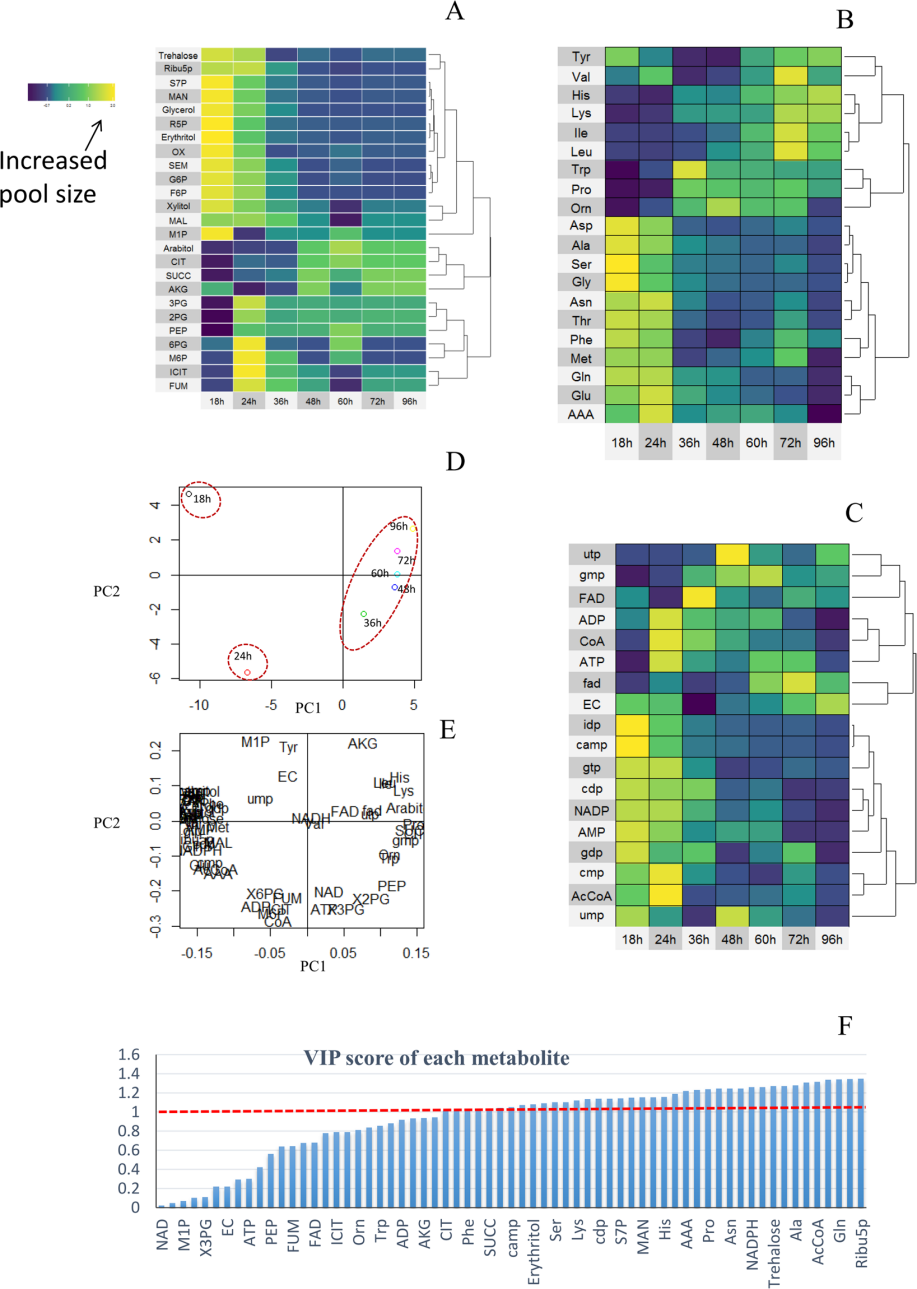
Data analysis of metabolomics at different fermentation phases (18 h, 24 h, 36 h, 48 h, 60 h, 72 h and 96 h. Heatmap of organic acid and sugar phosphates (**A**), amino acids (**B**), nucleotide and coenzymes (**C**), scores plot for samples in PCA analysis (**D**), loadings plot for metabolites in PCA analysis (**E**) VIP score of 69 intracellular metabolites calculated using PLS model (**F**). The detailed VIP scores for each metabolites can be found in Supplementary File 2.

**Figure 4:**
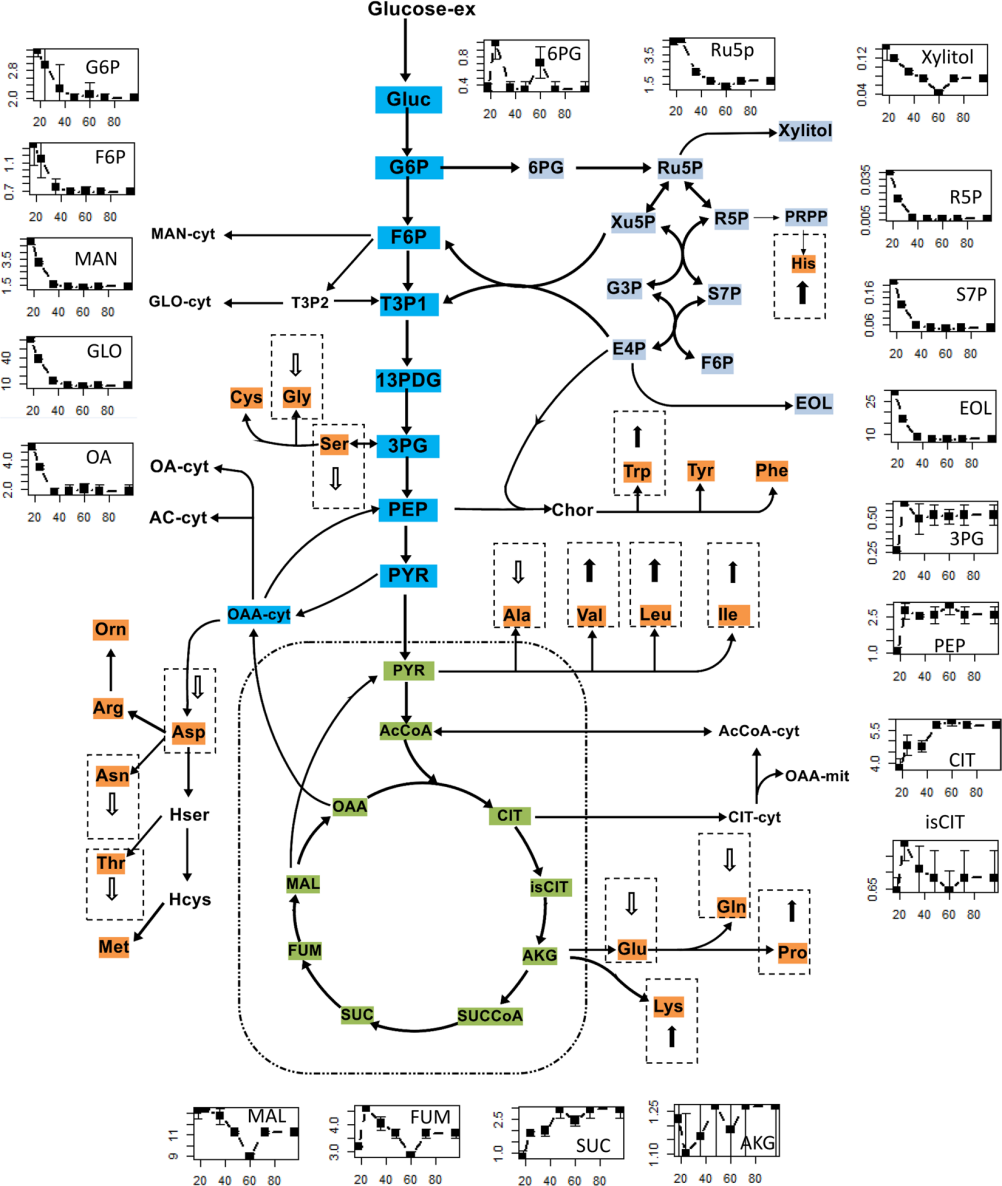
Schematic representation of the changes in the pool sizes of organic acids, sugar phosphates and amino acids during different fermentation phases onto the core carbon metabolism network. All metabolite pool sizes were determined in at least triplicate measurements. The fermentation time (h) is on the x-axis and the metabolites concentration (μmol/gDCW) is on the y-axis. The arrows in the small gridlines represent the increase or decrease for the pool sizes of intracellular amino acids during the oxygen limited phase compared to that in oxygen excess phase. The detailed profiles of intracellular amino acids pool sizes could be found in Supplementary File 1, Fig. S2.

In the first time, the pool sizes of 65 intracellular metabolites (amino acids, organic acids, sugar phosphates, nucleotides and coenzymes) from different fermentation phases were determined by LC-MS/MS or GC-MS. As shown in Fig. 3A–C, the pool sizes of most intracellular metabolites decreased sharply when *A. niger* entered into the oxygen limited phase. However, it was also found that some amino acids (like Tyr and Val) and organic acids (like SUCC and CIT) still accumulated over time (Fig. 4). The accumulation of organic acids was consistent with the high intracellular redox level under limited O_2_ supply^10,28^.

The changes in intracellular amino acid pool sizes during the oxygen limited phase exhibited two different tendencies (Figs 3B and 4). The pool sizes of Ala, Gly, Asp, Glu and Ser decreased sharply when the cells entered into the oxygen limited phase, while the pool sizes of Val, Leu, Ile and His increased significantly (see Supplementary File 1, Fig. S2), consistent with the extracellular accumulation of these amino acids (see Supplementary File 1, Fig. S3).

Furthermore, the principal component analysis (Fig. 3E) showed that all the samples can be categorized into three groups, which is difficult observed from physiological profiles (Fig. 2). On the other hand, the relation between changes in pool sizes of metabolites and q_O2_ was studied by partial least squares (PLS) analysis. The VIP of 45 metabolites was above 1 (Fig. 3F), indicating that the changes of most intracellular metabolites concentration were sensitive to the external environment perturbations.

### Systematic analysis of gene and typical transcription factors (TFs) expression pattern related to external environmental changes

**Figure 5:**
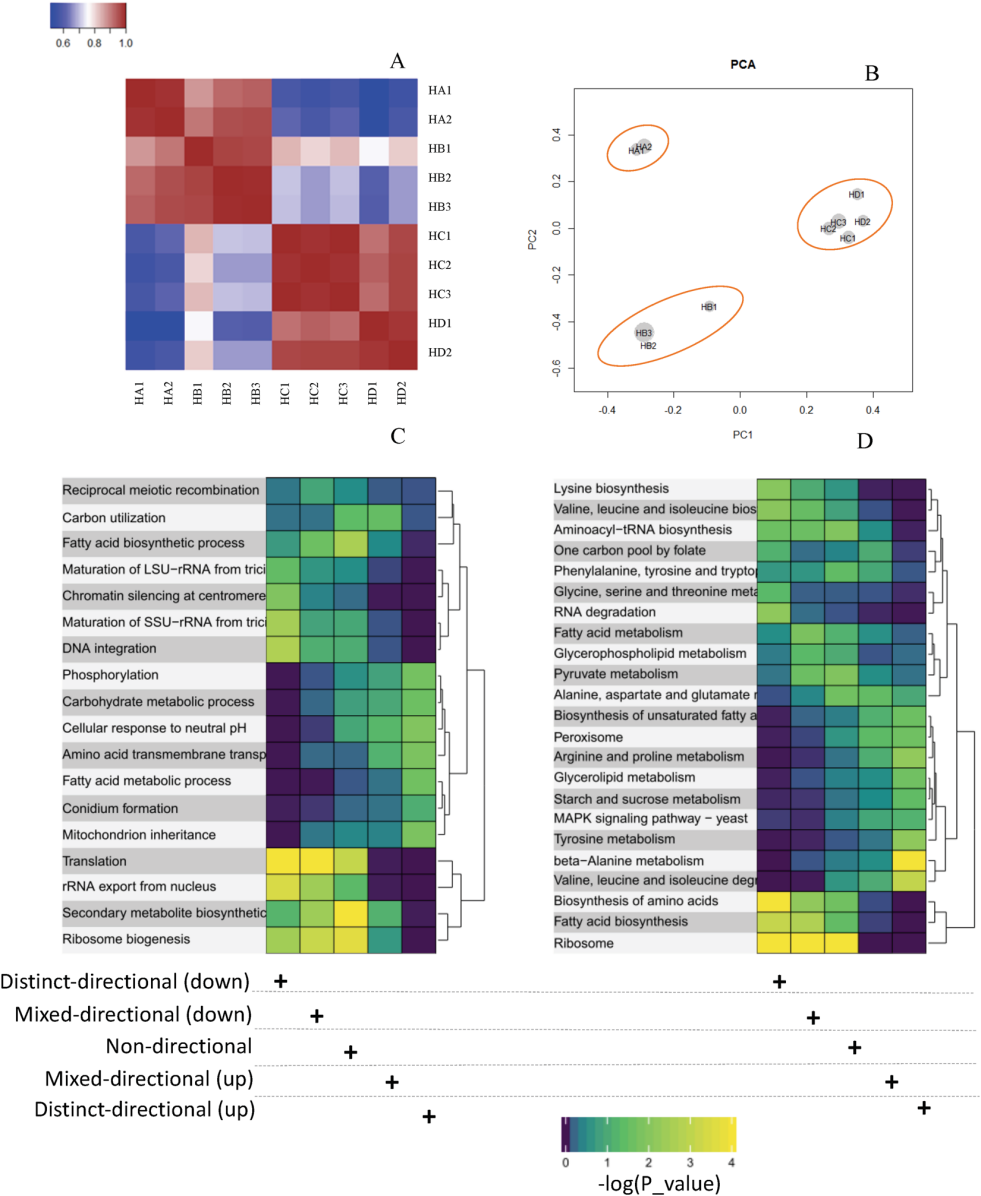
Gene set analysis of differentially expressed genes in the aerobic (16 h) and oxygen limited phases (42 h). Correlation coefficients of samples (**A**), scores plot for samples in PCA analysis using gene expression values (**B**), Gene set analysis based on GO function annotation (**C**) and KEGG pathway enrichment analysis (**D**). The non-directional class disregards the direction of change. The distinct-directional class takes direction of change into account. The mixed-directional class considers the up-regulated subset and the down-regulated subset of a gene set separately. Each subset is scored according to the proportion of significant genes.

The expression data of 10,445 genes from different fermentation phases (16 h, 24 h, 42 h and 66 h) was determined using RNA-seq. According to PCA analysis based on FPKM values of genes (Fig. 5B), it is shown that the replicate samples from the same time point could be clustered together. All the samples can be clustered into three groups, consistent with that using metabolomics analysis. To obtain the main metabolic characteristics of *A. niger* under oxygen limited environment, the gene expression pattern analysis along the fed-batch process was firstly carried out, followed by the gene set analysis of differentially expressed genes in two distinct fermentation phases (oxygen sufficient phase 16 h and oxygen limitation phase 42 h).

**Figure 6:**
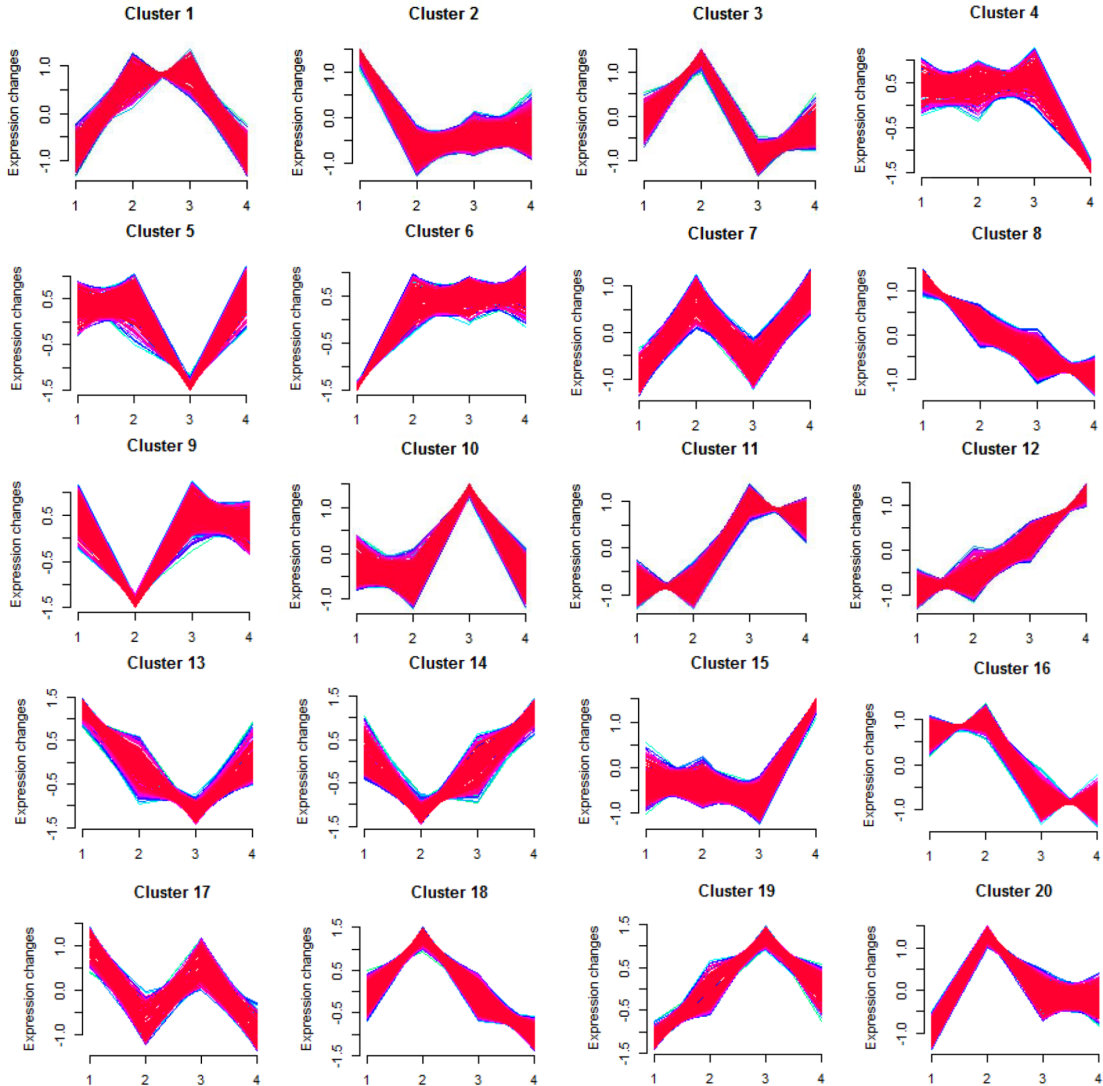
Clusters of expressed genes during different fermentation phases. The number of 1, 2, 3 and 4 in each small graph represents 16 h, 24 h, 42 h and 66 h respectively.

With the aid of the Mfuzz package^21^ based on the R language, those genes, with little expression (even no expression) at least two or more time points during the fermentation, were firstly removed and finally 6,662 genes were screened from the total 10,445 genes. Then the cluster analysis of gene expression profiles was conducted for the remaining 6,662 genes. The result showed that the expression pattern of all 6,662 genes could be divided into 20 clusters (Fig. 6). Among the 20 clusters, there exist several clusters in which the expression of genes was consistent to the changes of q_O2_ (Fig. 2G). For example, the expression of 408 genes in cluster 8 decreased with the decrease of q_O2_, while in cluster 12, the expression of 373 genes increased with the decrease of q_O2_. In addition, in cluster 2, the expression of 592 genes decreased sharply from the aerobic phase (16 h) to the transition phase (24 h), and remained stable in the mid and later oxygen limitation phases (42 h and 66 h). In contrast, 201 genes in cluster 6 showed an opposite tendency.

DAVID, an online gene function annotation tool^22^, was exploited to carry out gene function annotation and enrichment analysis of the genes from the up-regulation group (cluster 12 and cluster 6) (Table 1) and the down-regulation group (cluster 8 and cluster 2) (Table 2). There were about 423 transcription factors (TFs) in *A. niger* genome according to the *Aspergillus* Genome Database^29^. In the above cluster analysis, the expression of TFs in different clusters was extracted and studied. Cluster 12, where the gene expression was continuously up-regulated, contained 10 TFs, among which *flbA* (An02g03160, related to morphological development) and *riaA* (An16g05550, related to NADPH oxidation regulation) were included. Another TF, *brlA* (An01g10540), related to the formation of spores in cluster 2, was down-regulated under oxygen limited condition. The expression of 9 TFs in cluster 8 was significantly down-regulated. Among them, An12g00130, which is thought to play a role in regulating the mitochondrial respiratory chain complex IV biogenesis. Under oxygen limited conditions, the cell could decrease the biosynthesis of complex IV in the electron transport chain by down-regulating the expression of An12g00130.

**Table 1:** GO function enrichment analysis of genes from the up-regulation group.

Category	GO Number	Term	P-Value
**cluster 12**			
GOTERM_BP_FAT	GO:0006913	nucleocytoplasmic transport	0.001
GOTERM_BP_FAT	GO:0051169	nuclear transport	0.001
GOTERM_CC_FAT	GO:0005643	nuclear pore	0.002
GOTERM_CC_FAT	GO:0046930	pore complex	0.002
GOTERM_CC_FAT	GO:0005635	nuclear envelope	0.002
GOTERM_BP_FAT	GO:0046907	intracellular transport	0.024
GOTERM_CC_FAT	GO:0031967	organelle envelope	0.029
GOTERM_CC_FAT	GO:0012505	endomembrane system	0.030
GOTERM_CC_FAT	GO:0031975	envelope	0.032
GOTERM_BP_FAT	GO:0051170	nuclear import	0.034
GOTERM_BP_FAT	GO:0034504	protein localization in nucleus	0.034
GOTERM_BP_FAT	GO:0006606	protein import into nucleus	0.034
GOTERM_CC_FAT	GO:0016021	integral to membrane	0.035
GOTERM_CC_FAT	GO:0031224	intrinsic to membrane	0.040
GOTERM_MF_FAT	GO:0004857	enzyme inhibitor activity	0.050
INTERPRO		Hypoxia induced protein conserved region	0.057
GOTERM_BP_FAT	GO:0017038	protein import	0.076
SP_PIR_KEYWORDS		tricarboxylic acid cycle	0.077
GOTERM_BP_FAT	GO:0006979	response to oxidative stress	0.084
GOTERM_MF_FAT	GO:0004022	alcohol dehydrogenase (NAD) activity	0.088
**cluster 6**			
INTERPRO		Acyl-CoA oxidase/dehydrogenase, type1/2, C-terminal	0.012
INTERPRO		Acyl-CoA oxidase/dehydrogenase, central region	0.014
GOTERM_MF_FAT	GO:0003995	acyl-CoA dehydrogenase activity	0.015
GOTERM_MF_FAT	GO:0009055	electron carrier activity	0.034
GOTERM_BP_FAT	GO:0043087	regulation of GTPase activity	0.043
GOTERM_BP_FAT	GO:0051336	regulation of hydrolase activity	0.043
GOTERM_MF_FAT	GO:0008336	gamma-butyrobetaine dioxygenase activity	0.048
GOTERM_BP_FAT	GO:0006091	generation of precursor metabolites and energy	0.077
GOTERM_MF_FAT	GO:0030695	small GTPase regulator activity	0.095

**Table 2:** GO function enrichment analysis of genes from the down-regulation group.

Category	GO Number	Term	P-Value
**cluster 8**			
GOTERM_BP_FAT	GO:0006259	DNA metabolic process	0.009
GOTERM_BP_FAT	GO:0033554	cellular response to stress	0.011
GOTERM_BP_FAT	GO:0006260	DNA replication	0.013
GOTERM_MF_FAT	GO:0000036	acyl carrier activity	0.014
GOTERM_MF_FAT	GO:0016597	amino acid binding	0.014
GOTERM_MF_FAT	GO:0043176	amine binding	0.014
GOTERM_BP_FAT	GO:0006281	DNA repair	0.022
GOTERM_BP_FAT	GO:0006974	response to DNA damage stimulus	0.023
GOTERM_MF_FAT	GO:0004386	helicase activity	0.028
GOTERM_MF_FAT	GO:0031177	phosphopantetheine binding	0.035
GOTERM_MF_FAT	GO:0031406	carboxylic acid binding	0.049
GOTERM_MF_FAT	GO:0048037	cofactor binding	0.050
GOTERM_BP_FAT	GO:0019748	secondary metabolic process	0.067
GOTERM_BP_FAT	GO:0051276	chromosome organization	0.089
GOTERM_MF_FAT	GO:0016879	ligase activity, forming carbon-nitrogen bonds	0.096
GOTERM_MF_FAT	GO:0004842	ubiquitin-protein ligase activity	0.097
**cluster 2**			
GOTERM_BP_FAT	GO:0030163	protein catabolic process	0.047
GOTERM_CC_FAT	GO:0044430	cytoskeletal part	0.049
GOTERM_MF_FAT	GO:0008171	O-methyltransferase activity	0.055
GOTERM_BP_FAT	GO:0009057	macromolecule catabolic process	0.064
GOTERM_CC_FAT	GO:0015630	microtubule cytoskeleton	0.067
GOTERM_MF_FAT	GO:0000166	nucleotide binding	0.085
GOTERM_CC_FAT	GO:0005856	Cytoskeleton	0.096
GOTERM_MF_FAT	GO:0004386	helicase activity	0.097
GOTERM_MF_FAT	GO:0016405	CoA-ligase activity	0.098

The gene set analysis of differentially expressed genes at 42 h and 16 h was further performed by the Piano package^23^ based on the R language (Fig. 5C,D). The results showed that in order to adapt to the limited oxygen supply, the biosynthesis of fatty acids and secondary metabolites, ribosome biogenesis and translation were significantly down-regulated (P_value < 0.05), while the fatty acid catabolism was up-regulated, which can further be validated by changes in tendencies of genes expression from the related synthesis pathway (see Supplementary File 1, Fig. S6).

### Flux simulation based on newly updated GEMs

**Figure 7:**
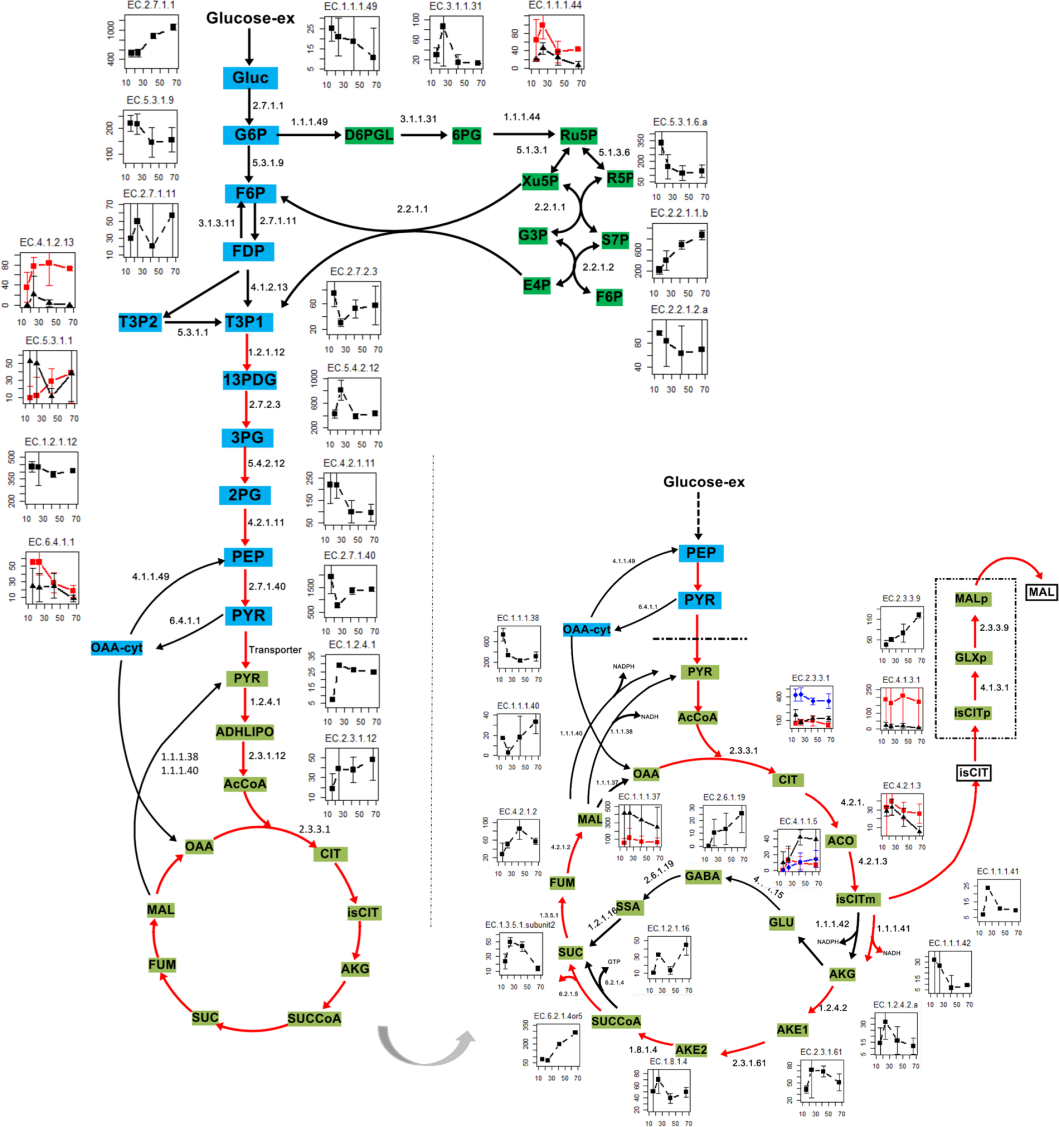
Trends of genes expression and relative flux of the EMP and PP and TCA pathways under different fermentation phases. The red arrows represent the reactions with an obvious increase in the relative flux predicted by iHL1210 in the oxygen limited phase. The fermentation time (h) is on the x-axis and the gene expression value (FPKM) is on the y-axis. The black and red lines in each small graph represent independent isogenies encoding the specific enzymes. The detailed flux distribution by pFBA at different time points could be found in Supplementary File 3.

To further investigate how the cell adapted to the external environmental changes, the flux distribution predicted by pFBA was exploited. The predicted μ and q_CO2_ are consistent with the measured values (see Supplementary File 3), indicating the good performances of iHL1210. The flux simulation using iHL1210^5^ showed that the relative fluxes through the EMP pathway increased when the cells entered into the oxygen limited phase (Fig. 7, Supplementary File 3). Consistent with the increased relative flux through EMP pathway, the expression of the corresponding genes was maintained at a stable level, such as fructose-bisphosphate aldolase (EC 4.1.2.13) and pyruvate dehydrogenase (EC 1.2.4.1, EC 2.3.1.12). In addition, the pool sizes of some amino acids from the aromatic and pyruvate families increased accordingly, which might provide precursors for enzyme production.

Flux simulation of GEMs iHL1210 showed that relative fluxes of the PP pathway decreased slowly along the fermentation (see Supplementary File 3), consistent with the reduction tendency in the gene expression values for glucose-6-phosphate dehydrogenase (EC 1.1.1.49). However, it was notable that gene expression of transketolase (EC 2.2.1.1) in the PP pathway was significantly up-regulated.

Consistent with higher pool sizes of organic acids, the flux simulation with iHL1210 showed that the relative flux through the TCA cycle was increased upon oxygen limitation, which was related to the lower demand for the anabolic metabolism from the cell growth. To maintain the redox balance, the cell metabolic activities were regulated in transcriptional level. Firstly, the expression of genes encoding the key enzymes of the TCA cycle, including citrate synthase (EC 2.3.3.1) and aconitate hydratase (EC 4.2.1.3), were down-regulated. Meanwhile, the expression of genes (EC 2.3.3.9 and EC 2.6.1.19) from the glyoxylate and GABA cycles were significantly up-regulated (Fig. 7). Consistent with this observation, the flux simulation indicated that the relative flux through the glyoxylate cycle increased (see Supplementary File 3).

## Discussion

### Cell metabolism based on gene expression pattern analysis under oxygen limited conditions

According to the cluster analysis in the Results section, there exist two obvious gene expression patterns, namely up-regulation (cluster 12 and cluster 6) and down-regulation (cluster 8 and cluster 2). Under oxygen limited conditions, the energy supply could become a main bottleneck for complex cell metabolic functions including membrane synthesis. The enrichment analysis of genes in cluster 6 revealed that the energy production was up-regulated during the oxygen limited phase, helping to relieve the shortage in energy supply. Genes in cluster 8 were enriched in the biological processes closely related to DNA unwinding, replication and transcription, indicating that oxygen limitation could weaken the transcription and translation to reduce energy demand. Parts of the genes in cluster 2 were also enriched in DNA unwinding, indicating that the DNA replication was obviously weakened, which was highly consistent with the decreased specific growth rate during the oxygen limited phase (Fig. 2H). Meanwhile, the synthesis of macromolecules slowed down and the expression of genes encoding degradation pathways of macromolecules (like proteins) was up-regulated accordingly (cluster 2) to strengthen the turnover of intracellular metabolites. According to the genome annotation^29^, transcription factor *flbA* in cluster 12 is the regulator of the G-protein signaling protein. It has been reported that the deletion of this gene displayed the phenotype of long thin mycelium without arthrospore, and promoted protein secretion^30^. In this study, the expression of *flbA* increased continuously, which might thicken the cell wall and hinder the protein secretion. Sterol regulatory element-binding proteins (SREBPs) are important TFs for filamentous fungi to adapt to an anaerobic environment as the decrease of the intracellular sterol concentration in anaerobic environment will activate the expression of SREBPs^31^. In *A. niger*, the corresponding genes are *srbA* (An03g05170) and *srbB* (An14g02540), respectively^32^. In this work, the expression of the former was down-regulated first and then was up-regulated (in cluster 14) while the expression of the latter was continuously elevated (in cluster 12), indicating that SREBPs are conserved for fungi to adapt to the oxygen limited environment.

### Potential advantages of the oxygen limited strategy used for enzyme production by *A. niger*

Similar to *P. pastoris*^17^, an appropriate oxygen limitation favors glucoamylase production by *A. niger*. However, the detailed mechanisms behind this phenomenon are not clear. Generally, there is an inverse correlation between protein production and cell growth^33^ as the protein synthesis usually needs the competitive precursors for the cell growth. During the oxygen limited phase, μ was lower than 0.02 h^−1^ (Fig. 2H). It could be speculated that the low specific growth rate can be a possible reason of high yield of glucoamylase due to the fact that once the growth was limited, more NADPH, NADH and precursors could be fluxed into synthesis of glucoamylase. Especially, the increased relative flux in the EMP pathway, along with the accumulation of amino acids from the pyruvate and aromatic families, is beneficial for protein synthesis. Furthermore, the transcription factors relating to sterol synthesis, one important composition of the cell membrane, was activated during the oxygen limitation, which possibly helped to maintain the integrity of the cell membrane^14^, favoring an efficient secretion of glucoamylase.

It has been reported that the exogenous addition of the limited amino acids could effectively alleviate the shortage in supply of amino acids, energy and reducing power^34^. In this work, the addition of Ala, Gly, Asp, Glu and Ser could obviously promote the enzyme production (see Supplementary File 1, Fig. S4). Coincidentally, these amino acids have a decreased tendency in intracellular pool sizes along with the fermentation. In combination with the fact that the four amino acids are the main compositions of glucoamylase (Table 3), it can be inferred that they might be the limiting precursors for the synthesis of the target protein, which provides new clues for metabolic engineering to promote enzyme production efficiency using molecular biology. It should be also noted that some amino acids, like His, Lys, Val, Ile, etc., were accumulated within the cell. For the accumulation of aromatic amino acids and part of the pyruvate family, a driver could be the increased flux through the EMP pathway. Moreover, the pool sizes of amino acids were closely related to the changes in the expression levels of genes from the corresponding pathways. Taking Ala and Gly as an example, the decrease in pool sizes of these two amino acids was accompanied with the observation of a prominent decrease in gene expression levels of the Ala and Gly synthesis pathways (see Supplementary File 1, Fig. S5), initially indicating that the pool sizes of amino acids were rigidly controlled. In terms of energy requirements, Lys, Met, Ile, Trp and His can be regarded as expensive amino acids^35^. It is reported that under environmental stress conditions the cell could secure some of the expensive amino acids while at the same time decreasing the pool sizes of the cheap amino acids^36^. As a result, when the environment changes again to more favorable conditions, the cell could realize fast growth by mobilizing the energy expensive amino acids. The precise mechanisms for the amino acids accumulation under oxygen limited conditions still need further experimental validation.

**Table 3:** Amino acid composition of glucoamylase.

animo acid	content	amino acid	content
Ala	10.17%	Met	0.47%
Cys	1.56%	Asn	3.91%
Asp	6.89%	Pro	3.44%
Glu	3.91%	Gln	2.66%
Phe	3.44%	Arg	3.13%
Gly	7.20%	Ser	13.77%
His	0.63%	Thr	11.58%
Ile	3.76%	Val	6.57%
Lys	2.03%	Trp	3.13%
Leu	7.51%	Tyr	4.23%

**Figure 8:**
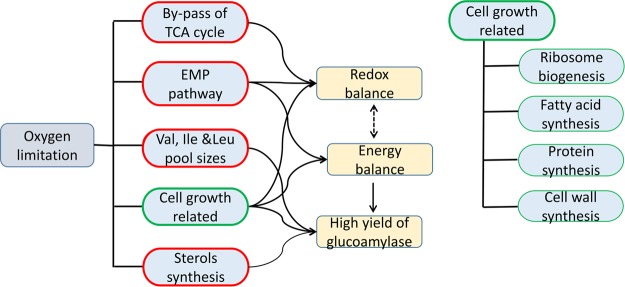
Possible coordinated regulation mechanisms for maintenance of the redox (energy) balance, as well as for the high enzyme production by *A. niger* upon oxygen limitation. The contents in red and green frames represent the up- and down-regulated metabolic activities, respectively.

The possible mechanisms for high yield of glucoamylase during the oxygen limited phase are summarized in Fig. 8.

### How the cell maintains redox and energy balance under hypoxic conditions?

Under hypoxic conditions, the ways for different species to maintain intracellular energy and redox balance may be different. Unlike fungal strains, *S. cerevisiae* and mammalian cells could regenerate NADH via the formation of ethanol and lactic acid to maintain the intracellular energy and redox balance. With ^13^C labeling experiments, the P/O ratio of *S. cerevisiae* was still found high under hypoxic conditions^37^, which indicates that the oxidative phosphorylation remained efficient. A high formation rate of ATP helps to promote the oxidative phosphorylation under hypoxic conditions^38^. Also, the oxygen limitation conditions could reduce leakage of protons and uncoupled respiration^38^. In this work, the marginal secretion of polyols and reduced organic acids was not enough to sustain the regeneration of NAD^+^. Therefore, it could be speculated that, similar to *A. fumigatus*^13^, oxidative phosphorylation is the main metabolic pathway for maintaining the intracellular balance of energy and redox. The transcriptomics and flux simulation indicated that the flux through the glyoxylate cycle was increased, reducing NADH formation from the TCA cycle, which helps to maintain the redox balance. In addition, as reported in the literature, during oxygen limited conditions, the strain could maintain the redox balance by the reduction of nitrate, as well as the secretion of branched amino acids^39^. As ammonium sulfate was used as nitrogen source in the work, the reduction of nitrate could be excluded. As for the secretion of branched amino acids, like Val, Ile, Leu, etc., they are slightly accumulated within the cell and the extracellular secretion was marginal, although still insufficient to maintain the intracellular redox balance. Therefore, we concluded that mainly the enforcements of glyoxylate cycle (the GABA shunt is also possible) and oxidative phosphorylation help to maintain the intracellular redox balance.

On the other hand, the oxygen limitation could lead to the shortage in supply of ATP and NADPH. To maintain the cell normal metabolic activities, the cell can reduce anabolism and strengthen the catabolism, which was validated by a decrease in the gene expression levels of the fatty acid synthesis pathway, as well as the increase in gene expression levels of the fatty acid catabolism pathway. The enforcement of the EMP pathway could increase the formation of ATP, alleviating the energy demand. As for NADPH supply, there are mainly three sources according to transcriptomics data: the PP pathway, ICIT + NADP =  > AKG + NADPH and MAL + NADP =  > PYR + NADPH. The gene expression levels of the former two sources were decreased while they were increased in the third source. With ^13^C labeling flux analysis^40^, it was found that in the *A. niger* high-producing strain, the flux through MAL + NADP = > PYR + NADPH was higher than that in the wild type strain. So it could be concluded that under oxygen limited conditions, MAL + NADP = > PYR + NADPH might be a potential gene target for metabolic engineering to provide more NADPH supply. The supposed metabolic regulation mechanisms for the cell to maintain the intracellular redox and energy balance can be found in Fig. 8.

## Conclusion

The multi-omics integrative analysis provides us new insights on the mechanisms of *A. niger* metabolic regulation under fed-batch process conditions for enzyme production. To maintain the intracellular redox and energy balance under hypoxic condition, the cell metabolism was regulated at different aspects. The pool sizes of most intermediate metabolites from the upper EMP and PP pathways decreased along the fermentation. Meanwhile the gene expression was reduced for the fatty acid and ribosome synthesis pathways accordingly to weaken the cell anabolic metabolism. On the contrary, the EMP pathway and glyoxylate pathway were activated, which can be validated by the association analysis of transcriptomics and fluxomics. The possible reasons for a high yield of glucoamylase during the oxygen limited phase can be summarized as follows. Firstly, the increased relative flux through the EMP pathway could provide more precursors for enzyme synthesis. Secondly, the down regulations in fatty acid and ribosome biogenesis could also channel more precursors towards glucoamylase synthesis. Thirdly, the up-regulation in gene expression for sterols synthesis might favor the enzyme secretion. The multi-omics integrative analysis illustrated, in a systematic view, the potential of an oxygen limited strategy used in the industrial fed-batch fermentation. Furthermore, a rational optimization of the *A. niger* metabolic network in terms of precursors and NADPH supply, as well as re-balancing the NADH and sterol biosynthesis may further help *A. niger* adapt to hypoxic conditions, as well as improvement of the enzyme productivity.

## Electronic supplementary material

Supplementary file 1

Supplementary file 2

Supplementary file 3

## Data Availability

All data generated or analysed during this study are included in this article (and its Supplementary Information Files).
