# Supplementary material for: Multi-omics integrative analysis with genome-scale metabolic model simulation reveals global cellular adaptation of Aspergillus niger under industrial enzyme production condition
Source: Sci Rep. 2018 Sep 26;8:14404. doi: 10.1038/s41598-018-32341-1 (PMC6158188; doi:10.1038/s41598-018-32341-1)
Supplement: Supplementary file 1 [file 41598_2018_32341_MOESM1_ESM.pdf]

Multi-omics integrative analysis with genome-scale metabolic model simulation reveals global cellular adaptation of Aspergillus niger under industrial enzyme production condition

Hongzhong Lu[a*], Weiqiang Cao[a*], Xiaoyun Liu, Yufei Sui, Liming Ouyang[a**], Jianye Xia[a], Mingzhi Huang[a], Yingping Zhuang[a], Siliang Zhang[a], Henk Noorman[b] ,Ju Chu[a**]

List of supplementary figures

Figure S1 Profiles of the main by-products secreted by *A. niger* along the fed-batch fermentations. OX: oxalic acid, CIR: citric acid, Xyl: xylitol, Ery: erythritol, Man: mannitol.

Figure S2 Profiles of metabolits pool size of amino acids during different fermentation phases of *A. niger*. All metabolite pool sizes were measured in at least triplicate measurements. The fermentation phase (h) is on horizontal axis

Figure S3 Changes in concentrations of extracellular amino acids during different fermentation phases of *A. niger*. The fermentation phase (h) is on horizontal axis and the concentration of amino acids (μmol/L) is on the vertical.

Figure S4 Effects of addition of exogenous amino acids on glucoamylase production (A: Enzyme concentration; B: Enzyme yield) by *A. niger*. All enzyme activities were measured in at least triplicate measurements.

Figure S5 Changes in tendencies of genes expression from synthesis pathway of different amino acids under varied fermentation phases. The fermentation phase (h) is on x- axis and the gene FPKM value is on the y-axis.

Figure S6 Changes in tendencies of genes expression from synthesis pathway of Nucleotide sugars (A), Chitin biosynthesis (B) and glycerolipid metabolism(C). The fermentation phase (h) is on x- axis and the gene FPKM value is on the y-axis.

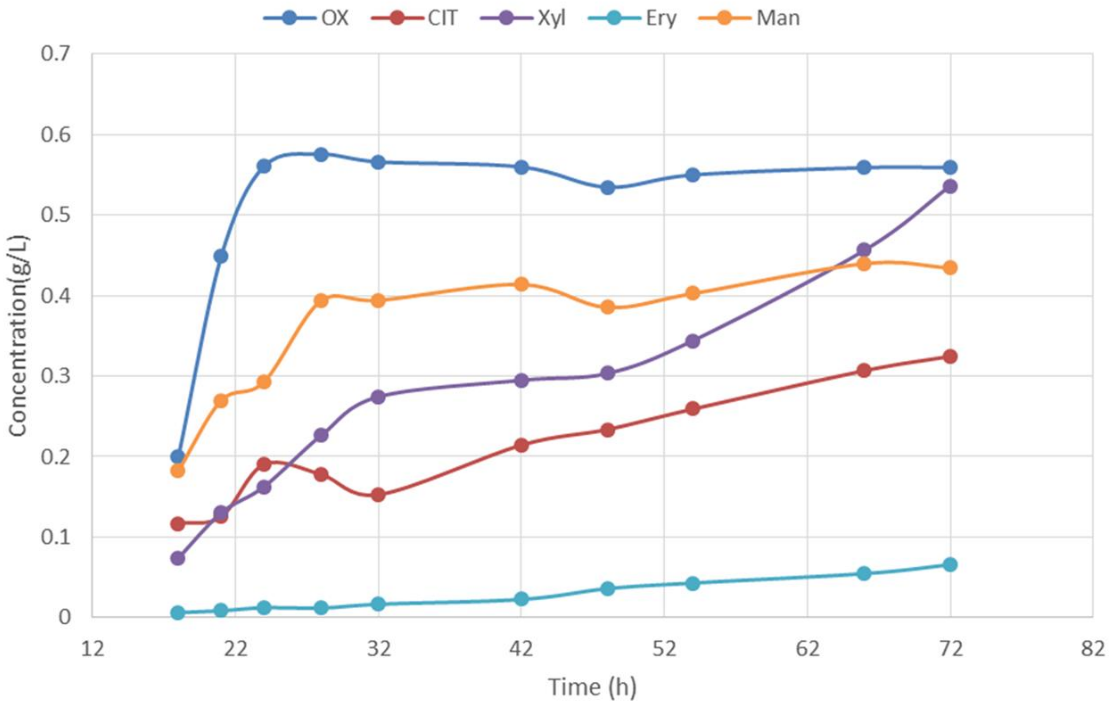

Figure S1 Profiles of the main by-products secreted by *A. niger* along the fed-batch fermentations. OX: oxalic acid, CIR: citric acid, Xyl: xylitol, Ery: erythritol, Man: mannitol.

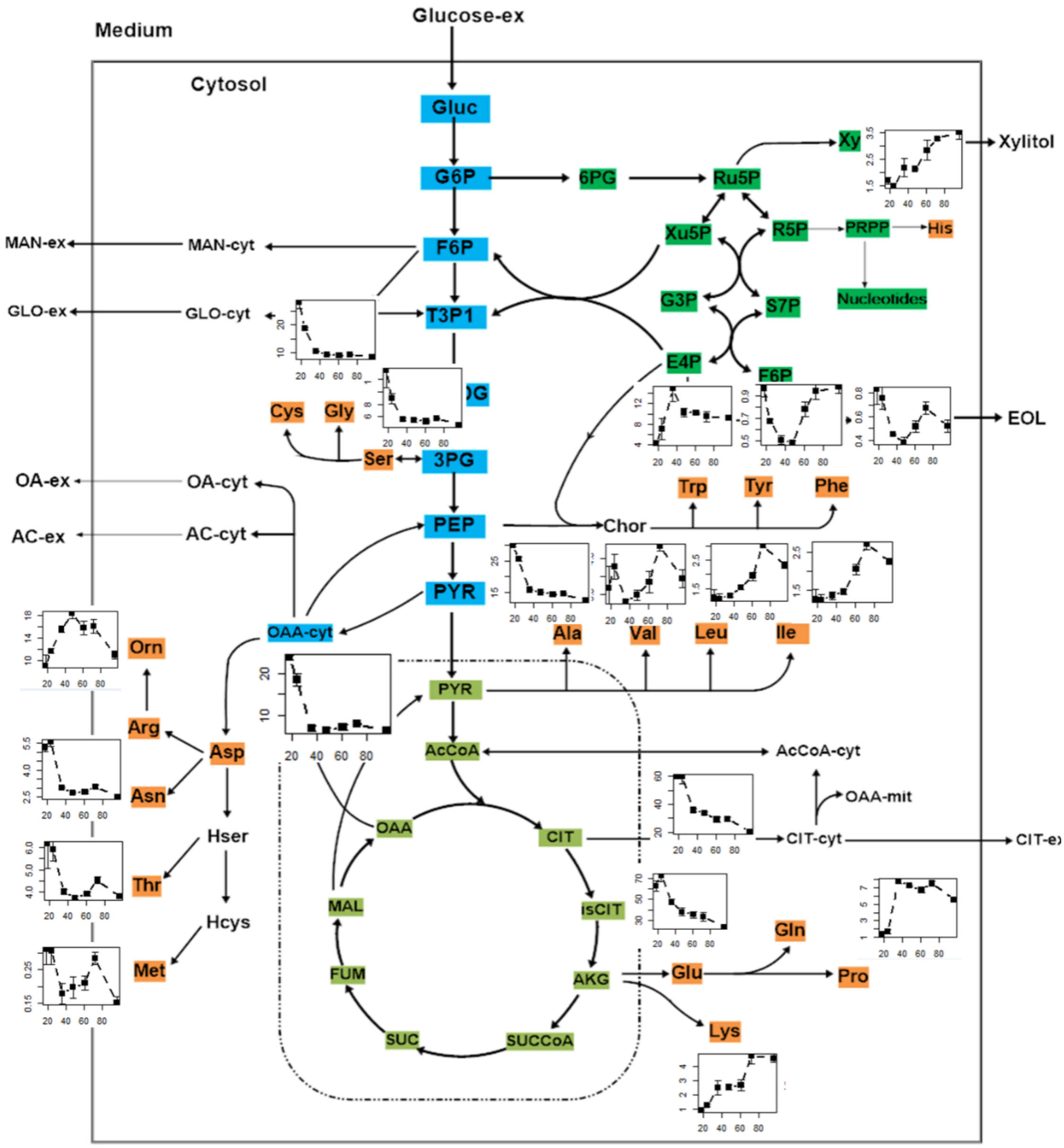

Figure S2 Profiles of metabolits pool size of amino acids during different fermentation phases of *A. niger.* All metabolite pool sizes were measured in at least triplicate measurements. The fermentation phase (h) is on horizontal axis and the pool sizes of amino acids (μmol/gBiomass) is on the vertical.

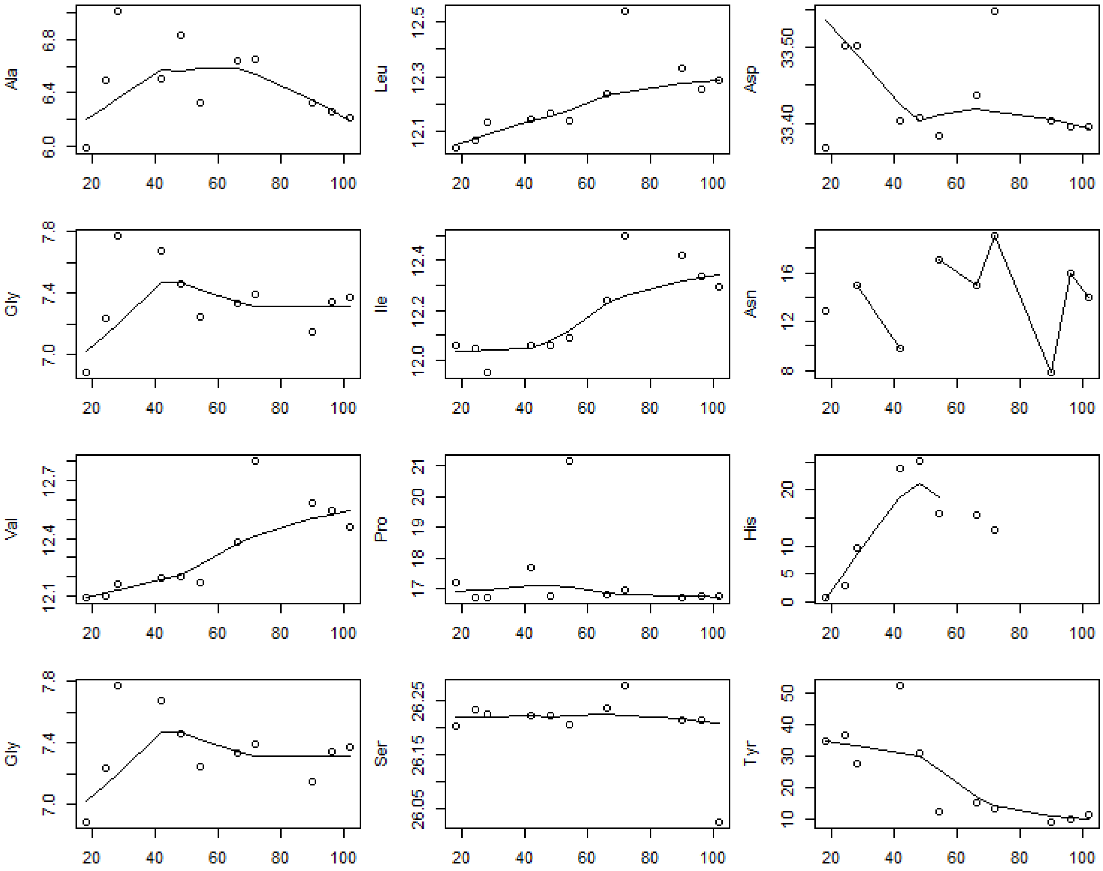

Figure S3 Changes in concentrations of extracellular amino acids during different fermentation phases of *A. niger*. The fermentation phase (h) is on horizontal axis and the concentration of amino acids (μmol/L) is on the vertical.

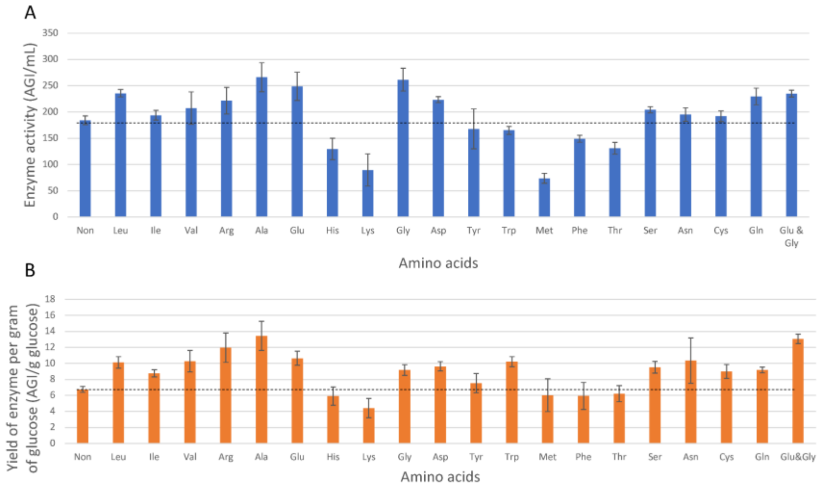

Figure S4 Effects of addition of exogenous amino acids on glucoamylase production (A: Enzyme concentration; B: Enzyme yield) by *A. niger*. All enzyme activities were measured in at least triplicate measurements.

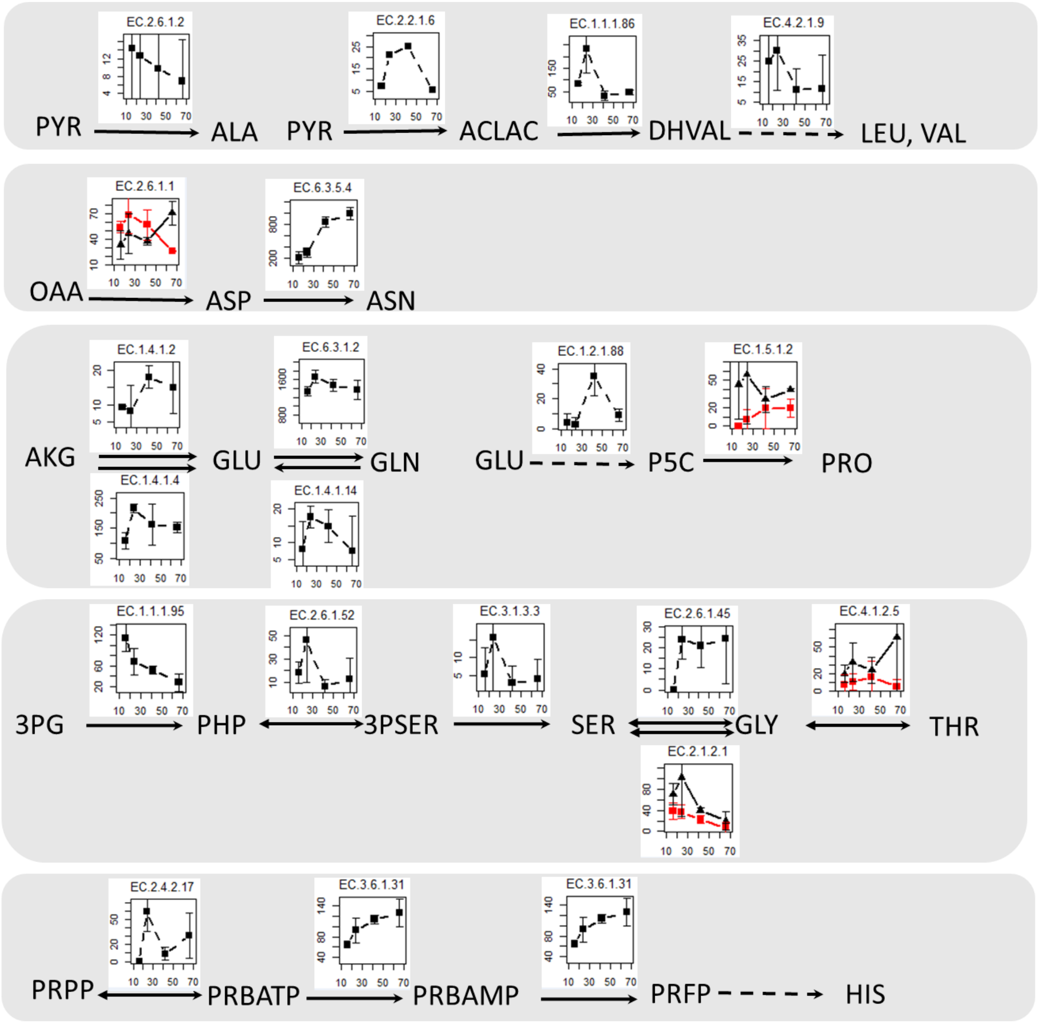

Figure S5 Changes in tendencies of genes expression from synthesis pathway of different amino acids under varied fermentation phases. The fermentation phase (h) is on x- axis and the gene FPKM value is on the y-axis.

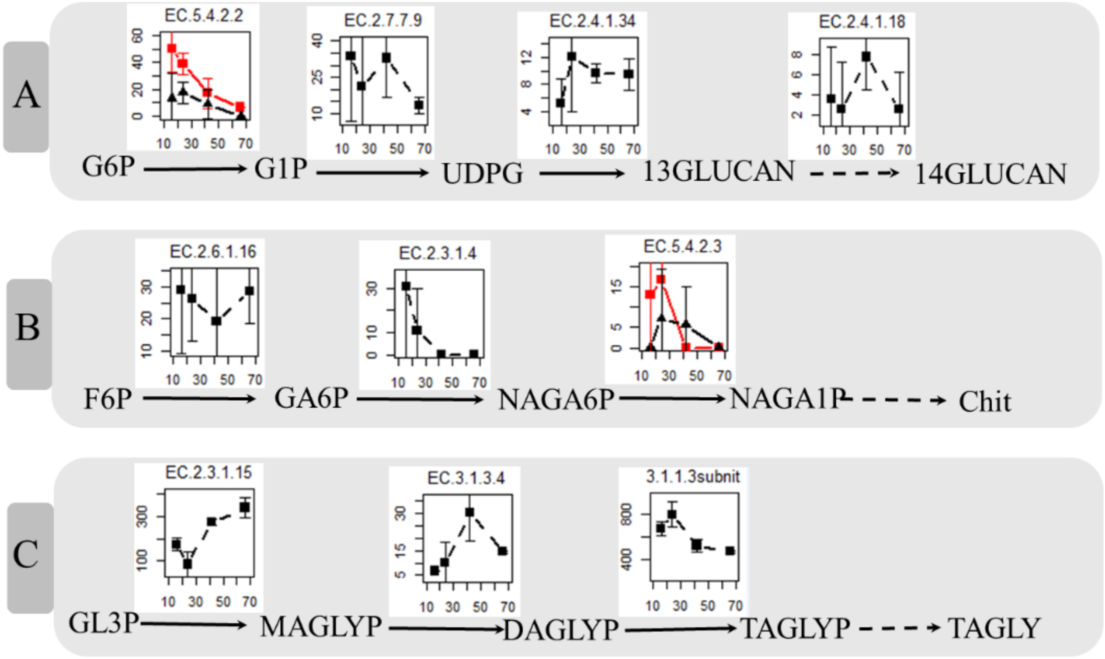

Figure S6 Changes in tendencies of genes expression from synthesis pathway of Nucleotide sugars (A), Chitin biosynthesis (B) and glycerolipid metabolism(C). The fermentation phase (h) is on x- axis and the gene FPKM value is on the y-axis.
